# Design and Implementation of a Biomimetic Underwater Robot Propulsion System Inspired by Bullfrog Hind Leg Movements

**DOI:** 10.3390/biomimetics10080498

**Published:** 2025-07-30

**Authors:** Yichen Chu, Yahui Wang, Yanhui Fu, Mingxu Ma, Yunan Zhong, Tianbiao Yu

**Affiliations:** 1 School of Mechanical Engineering and Automation, Northeastern University, Shenyang 110819, China; 2310088@stu.neu.edu.cn (Y.C.); 2300457@stu.neu.edu.cn (Y.W.); 2300370@stu.neu.edu.cn (Y.F.); 2400474@stu.neu.edu.cn (Y.Z.); tbyu@me.neu.edu.cn (T.Y.); 2 Beijing Lindong Bionic Science and Technology Limited Company, Beijing 100080, China

**Keywords:** bionic underwater robot, bullfrog hind legs, multi-linkage mechanism, motion capture

## Abstract

Underwater propulsion systems are the fundamental functional modules of underwater robotics and are crucial in intricate underwater operational scenarios. This paper proposes a biomimetic underwater robot propulsion scheme that is motivated by the hindlimb movements of the bullfrog. A multi-linkage mechanism was developed to replicate the “kicking-and-retracting” motion of the bullfrog by employing motion capture systems to acquire biological data on their hindlimb movements. The FDM 3D printing and PC board engraving techniques were employed to construct the experimental prototype. The prototype’s biomimetic and motion characteristics were validated through motion capture experiments and comparisons with a real bullfrog. The biomimetic bullfrog hindlimb propulsion system was tested with six-degree-of-freedom force experiments to evaluate its propulsion capabilities. The system achieved an average thrust of 2.65 N. The effectiveness of motor drive parameter optimization was validated by voltage comparison experiments, which demonstrated a nonlinear increase in thrust as voltage increased. This design approach, which transforms biological kinematic characteristics into mechanical drive parameters, exhibits excellent feasibility and efficacy, offering a novel solution and quantitative reference for underwater robot design.

## 1. Introduction

The improvement of marine exploration has led to a significant increase in the utilization of underwater robots for deep-sea resource exploitation, ecological environment monitoring, and intricate underwater operating scenarios [1,2]. The propulsion system, as a fundamental functional module [3], directly influences the robot’s endurance [4], maneuverability [5], and operational efficiency [6]. Although conventional propeller-driven propulsion systems provide significant efficiency, they are constrained by drawbacks including elevated noise levels and inadequate maneuverability [7]. In recent years, biomimicry has yielded novel insights into the design of underwater robots. By emulating the locomotion mechanics of aquatic organisms [8,9,10,11], propulsion effects characterized by low disturbance and high stability can be attained. Wright and others [12] were motivated by the American eel to develop a modular magnetic coupling biomimetic autonomous underwater robot. Utilizing permanent magnet synchronous magnetic coupling to link adjacent modules, the research accomplished a traveling wave motion that emulates the swimming of a body tail fin, providing benefits such as modular flexible configuration, dependable waterproof sealing, and secure module decoupling under extreme conditions. Bianchi et al. [13] created a biomimetic underwater robot inspired by the bullhead fish, implementing a three-independent-mechanism drive scheme for pectoral fin undulations. By optimizing the four-bar mechanism and conducting CFD dynamic analysis, the research successfully simulated the bullhead fish’s traveling wave propulsion, providing benefits in flexible maneuverability and synchronous control algorithms. Xiao Xiong et al. [14] created a reconfigurable biomimetic jellyfish robot, drawing inspiration from jellyfish and fish skeletal structures, utilizing soft hydraulic biomimetic actuators for propulsion. By independently regulating the mobility, grasping, and buoyancy control modules through hydraulic and pneumatic systems, the robot attains underwater mobility and grasping capabilities. Sameh and Elhenidy [15] created a biomimetic remote-controlled underwater robot inspired by cetaceans, characterized by a mechanical design that emulates the motion of whale flippers and tails, and incorporates the YOLOv12 model for ichthyological detection, thereby enhancing biological recognition and environmental adaptability in marine exploration. Soto et al. [16] developed a soft robotic fish inspired by aquatic species, featuring distributed musculature and integrated motion sensing. The concept integrates HASEL artificial muscles with strain sensors to maximize propulsion across various resonance frequencies and improve traveling wave motion. Ding and others [17] developed a biomimetic flapping underwater robot inspired by green sea turtles. Through experimental studies on its linear acceleration, turning maneuverability, and pitch-up/down motion performance, they found that flapping frequency affects the nonlinear growth of acceleration and speed, turning ability is determined by the total thrust and thrust ratio coupling of the left and right foils, and the balance angle can control diving speed, demonstrating excellent maneuverability.

Research in biomimetic underwater robotics has predominantly concentrated on the horizontal oscillatory propulsion mechanisms of standard aquatic species, particularly fish [18,19]. Frogs, as a specialized group within the amphibian class, demonstrate distinct biological advantages due to their hindlimb propulsion system characterized by vertical oscillation [20,21]. In contrast to fish and other aquatic organisms, the hind limbs of frogs produce an effective vortex ring effect in low-Reynolds-number flow environments [22] through the synchronized “kicking-retraction” actions of various joints [23], combining explosive thrust generation with accurate maneuverability, rendering them especially adept for near-bottom activities in intricate terrains. In recent years, researchers have developed submerged biomimetic robots modeled after frogs. Wang [24] and colleagues developed an adaptive flipper based on origami structures, utilizing foldable units and flexible connections to achieve adaptive motion: automatically unfolding to enhance thrust during the propulsion phase and autonomously contracting to diminish drag during the recovery phase. However, relying on passive deformation makes it impossible to actively control the coordinated movement of the frog’s hind limbs, resulting in low movement biomimicry. Furthermore, the origami structure has weak load-bearing capacity and cannot generate sufficient thrust. Pan et al. [25] developed a rope-driven, link-type frog swimming robot, inspired by frogs, employing a rope-driven link-type limb propulsion mechanism to facilitate multi-directional controllable movement, thereby validating the structural design and control rationale. However, rope-driven systems require multiple servo motors to work together, which makes them complex, and the elastic deformation of the rope can cause movement lag, affecting the coordination accuracy of the joint angles. Fan et al. [26,27] created a jointed pneumatic soft-driven robotic frog, drawing inspiration from real frogs, utilizing pneumatic soft actuators to replicate the movements of frog limbs. The research developed limb movement patterns using kinematic models, integrating them with a control system for miniaturization. However, the compressibility of gases causes drive response lag, low joint angle control accuracy, nonlinear deformation of soft drives under load, and unstable thrust output. Soomro et al. [28] created a fully 3D-printed multi-material soft biomimetic frog robot inspired by semi-aquatic frogs. The robot utilizes a multi-layer structural design that integrates shape memory alloys with ultra-flexible materials, enabling coordinated underwater locomotion via a dual-thrust generation mechanism, thus demonstrating the viability of soft robots in biomimetic propulsion. However, SMA’s drive depends on temperature changes, and its fully soft structure lacks rigid support, making it difficult to match the explosive propulsion of real frogs and resulting in insufficient long-term stability.

The bullfrog was chosen as the biomimetic model for two reasons: first, its status as a commonly farmed economic species enables the collection of kinematic data via standardized studies. This species is classified within the genus Hyla, exhibiting hindlimb muscle mass that constitutes over 35% of its body weight, tibia–fibula extension angles of 180° [29]. The optimized webbing fluid-acting surface area is large, and its resistance-based hindlimb propulsion mechanism results in a propulsion efficiency that is 22% higher than that of similarly sized small freshwater teleost fish using lift-based tail fin oscillation propulsion, making it a typical biological prototype in biomimetic mechanism design [30]. This work employed motion capture technology [31] to examine the locomotion of the bullfrog in aquatic environments. It developed a multi-jointed linkage mechanism to replicate the movement of a bullfrog’s hindlimbs in water. The device can simulate the movement of bullfrog hindlimbs using a single motor, addressing issues of complex coordination among multiple drive sources and response lag. The innovative design of a single-sided skeletal silicone fin actively achieves “expanding to increase surface area during propulsion and contracting to reduce resistance during recovery,” significantly improving propulsion efficiency compared to passive adjustments. This is of great significance for the effective design of underwater biomimetic robots and the advancement of underwater biomimetic propulsion technology.

## 2. Analysis of Bullfrog Movement

### 2.1. Experimental Protocol Design

**Figure 1:**
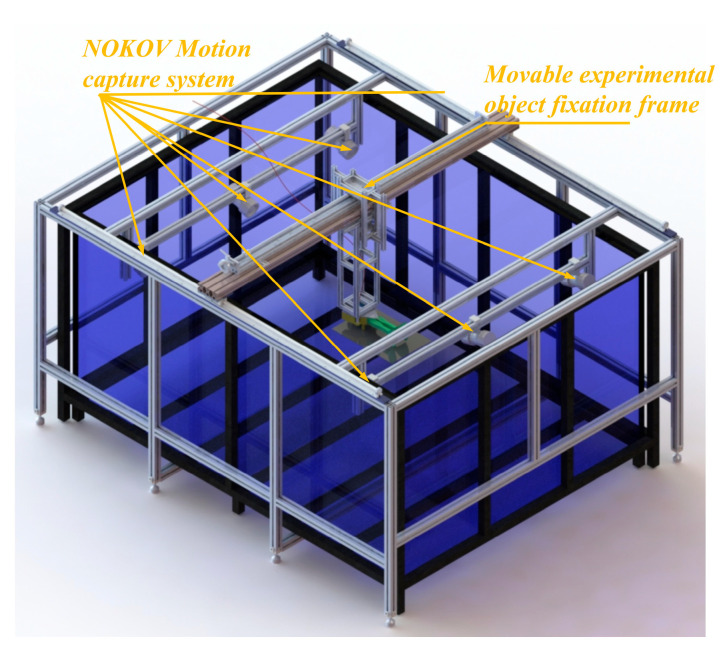
Schematic diagram of the motion capture experimental platform.

In order to acquire more precise biological data on the bullfrog for the development of a bionic bullfrog hindlimb propulsion system, a 6-month-old male bullfrog weighing 1 kg was acquired from an edible bullfrog supplier, and the movement data of the bullfrog were subsequently collected and analyzed. In order to ascertain the movement parameters of the bullfrog in water, a motion capture experiment was implemented. The experimental platform is equipped with a transparent glass pool that is 2000 mm in length, 1500 mm in width, and 1000 mm in height, as shown in Figure 1. Black-painted stainless steel square tubes are utilized to reinforce the pool’s sides and bottom. The NOKOV Motion Capture System is employed to conduct motion capture investigations, utilizing a passive marker-based optical capture solution. Six NOKOV underwater motion capture cameras are installed on the experimental platform, with one camera installed at each endpoint and midpoint along the two shorter sides of the tank. In order to prevent measurement errors caused by inter-medium refraction and achieve precise capture and analysis of the experimental subject’s motion posture in water, NOKOV’s underwater motion capture cameras were chosen, as the difference in refractive indices between air and water can affect the accuracy of motion data collection. The camera lens and active light source are fully submerged in water. The experimental subject’s fixed position in the water is configured to be adjustable in order to facilitate the installation of the experimental subject’s fixation components and to capture the movement of marker points by as many motion capture cameras as feasible during motion capture experiments. Tracks are installed along the long sides of the pool using 40 mm × 40 mm European standard aluminum profiles. Movable crossbeams, which are constructed from 40 mm × 80 mm European standard aluminum profiles, are mounted on the tracks. The crossbeam is equipped with a connection platform that can be adjusted in position using a hand-crank screw shaft and tracks. The connection platform is connected to the vertical support structure that secures the experimental subject. The marked points on the experimental subject can be captured by as many motion capture cameras as possible by manually adjusting the screw rod to alter the position of the movable experimental object fixation frame.

**Figure 2:**
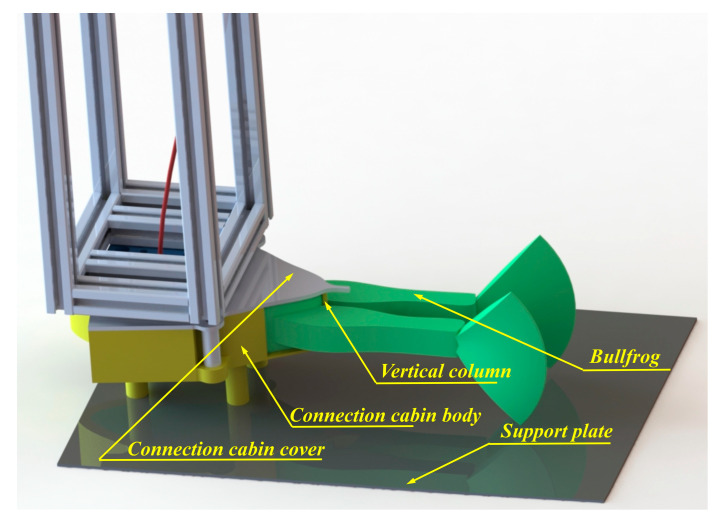
Schematic diagram of bullfrog fixation.

Since bullfrogs are natural organisms with smooth skin, it is difficult to secure them to the movable experimental object fixation frame using conventional methods. As shown in Figure 2, to prevent injury to the bullfrog during experiments, a connection chamber was tailored to the bullfrog’s body dimensions to accommodate its upper body. The connection chamber was manufactured using 3D printing technology. The connection chamber consists of a chamber lid and chamber body, allowing the bullfrog to be securely positioned within the chamber, with its hind limbs extending separately through openings at the rear end of the chamber. At the opening at the end of the connection chamber, a vertical column was designed to divide the opening into two equal-sized left and right sections, allowing the bullfrog’s left and right legs to extend separately while also preventing the bullfrog from slipping out of the opening due to movement. To more accurately collect movement data from each part of the bullfrog’s hind limbs during movement, a black matte-finished support plate was installed at the bottom of the connection chamber, keeping the bullfrog’s hind limbs’ movements above the plate.

### 2.2. Experimental and Test Data

**Figure 3:**
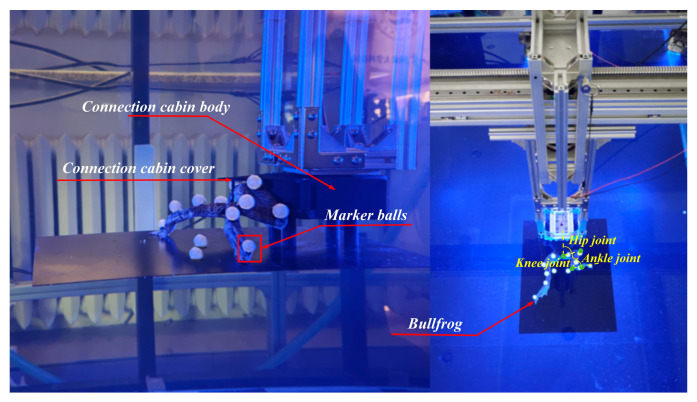
Schematic diagram of the motion capture experiment on a real bullfrog.

**Figure 4:**
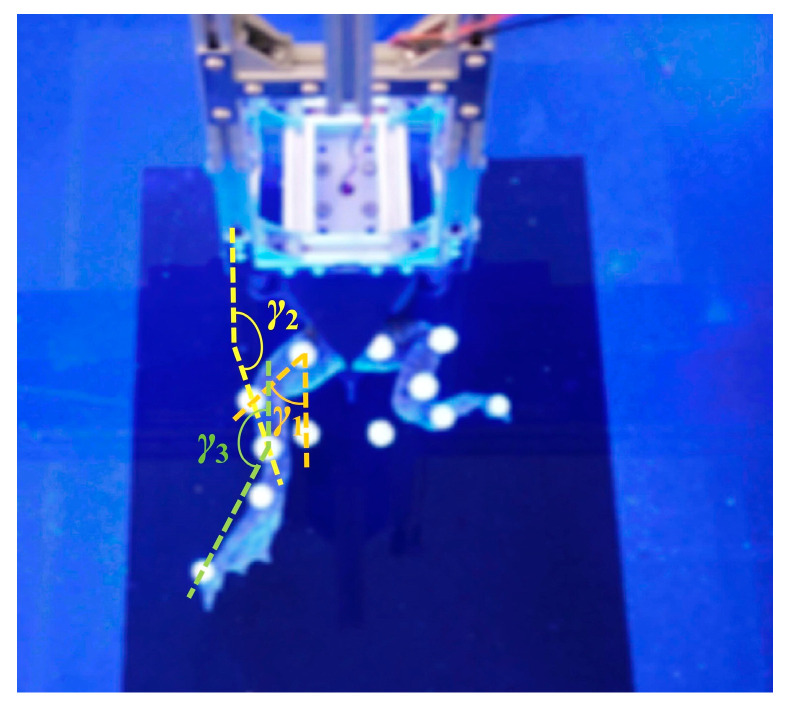
Schematic diagram of the motion capture angle measurement for a real bullfrog.

**Figure 5:**
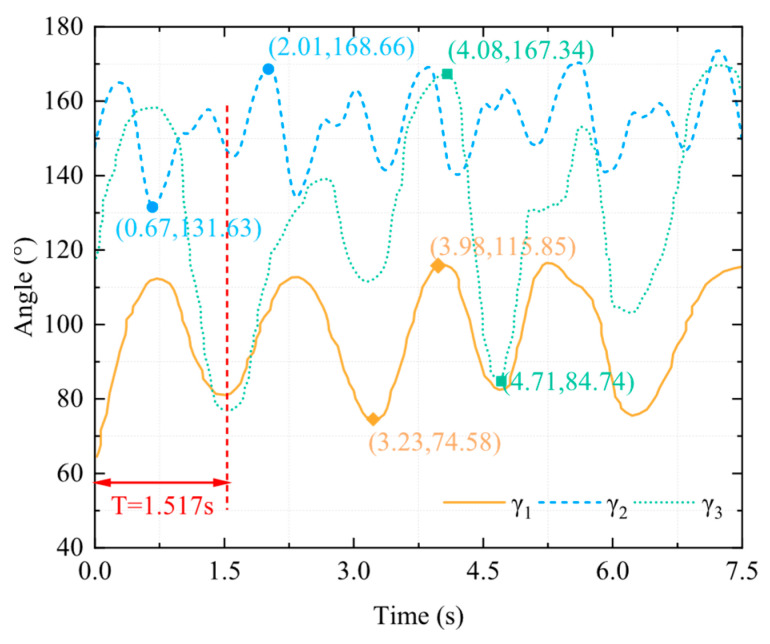
Motion capture data chart for a real bullfrog.

The connection chamber secured the bullfrog to the movable experimental object fixation frame, as shown in Figure 3. Rubber bands were employed to secure the marker balls around each segment of the bullfrog’s rear limbs, thereby ensuring that they are spaced appropriately and kept as far away from the bullfrog’s joints as possible, reducing the impact of the marker balls on the bullfrog’s movement. Each of the bullfrog’s hind extremities was adorned with five marker balls. Two marker spheres were positioned between the knee joint and ankle joint, one marker ball was positioned near the hip joint between the hip joint and knee joint, and two marker balls were positioned between the ankle joint and the tip of the foot webbing. The motion capture system was used to measure the angles *γ*_1_, *γ*_2_, and *γ*_3_ between the thigh, lower limb, and webbed foot relative to the vertical direction of the body in real time, as shown in Figure 4. The bullfrog was allowed to move freely. During the experiment, a rigorous screening process was implemented to guarantee the accuracy and reproducibility of the data. Only valid data were retained when the bullfrog was at its maximal movement angle. To acquire reliable samples, five replicate experiments were implemented. The analysis demonstrated that the three angles *γ*_1_, *γ*_2_, and *γ*_3_ underwent modifications during the bullfrog’s hindlimb movement, as shown in Figure 5. The bullfrog’s biological movement cycle is 1.517 s, as indicated by the analysis. The angle *γ*_1_ between the bullfrog’s thigh and the vertical direction of the bullfrog’s body is estimated to vary between 74.58° and 115.85°. The peak standard deviation is ±3.2°, and the valley standard deviation is ±2.8°. The angle *γ*_2_ between the bullfrog’s lower leg and the vertical direction of the bullfrog’s body is estimated to vary between 131.63° and 168.66°. The peak standard deviation is ±4.1°, and the valley standard deviation is ±3.5°. The angle *γ*_3_ between the bullfrog’s foot webbing and the vertical direction of the frog’s body varies between 84.74° and 167.34°. The peak standard deviation is ±2.5°, and the valley standard deviation is ±2.1°. Among them, the marked data points in Figure 5 represent the extreme points of the change in angle *γ*_1_, *γ*_2_, and *γ*_3_ within the test period.

## 3. Mechanical Design of Bionic Bullfrog Hindlimb

**Figure 6:**
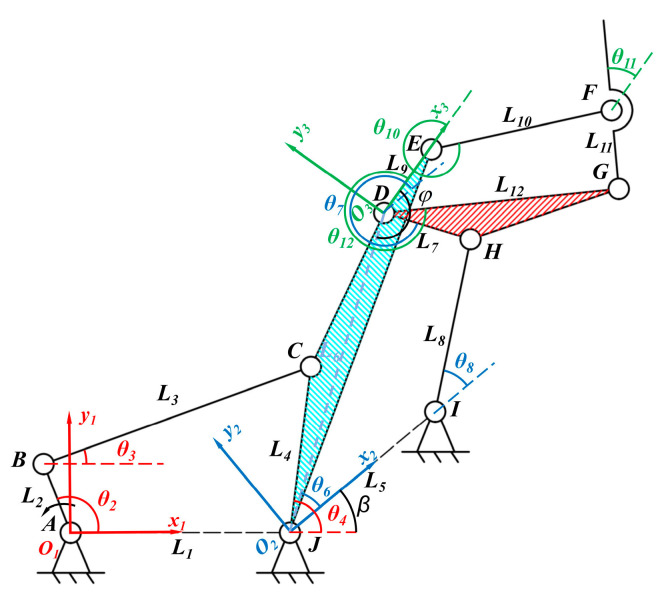
Schematic diagram of the bionic bullfrog mechanism and the establishment of the relevant coordinate system.

Particularly critical is the hind limb mechanism design in the context of bionic bullfrog hind limbs. The bullfrog, a member of the Hyla genus, is distinguished from other frogs by its fibula’s ability to extend to nearly 180°. In order to replicate the movement of the three segments of the bullfrog’s hindlimb, a multi-jointed linkage mechanism is constructed by serializing three four-bar mechanisms, as shown in Figure 6. The third-stage four-bar mechanism mimics the bullfrog’s thigh and lower leg by utilizing links *DE* and *EF*, thereby simulating the complete extension of the bullfrog’s knee joint. The first-stage four-bar linkage is chosen as a crank–rocker mechanism to further simplify control complexity and mitigate discontinuous mechanism movement caused by rotational direction changes. The active component is driven by a gear that is fixed to it and meshes with the motor output shaft gear. The second-level four-bar mechanism and the third-level four-bar mechanism are both double rocker mechanisms. The swing rod of the first-level four-bar mechanism drives the motion of the second-level four-bar mechanism, while the connecting rod between the two swing rods of the second-level four-bar mechanism drives the motion of the third-level four-bar mechanism. In the multi-jointed linkage mechanism, the *JCDE* link simulates the bullfrog’s thigh, the *EF* link simulates the bullfrog’s lower leg, and the *GF* link replicates the bullfrog’s flipper.

Figure 6 presents the schematic diagram of the bionic bullfrog mechanism and the establishment of the corresponding coordinate systems. The first-level four-bar mechanism, the second-level four-bar mechanism, and the third-level four-bar mechanism establish coordinate systems *x*_1_*O*_1_*y*_1_, *x*_2_*O*_2_*y*_2_, and *x*_3_*O*_3_*y*_3_, respectively. The theoretical configuration of the first-stage mechanism of the bionic bullfrog’s hind limb is a four-bar mechanism composed of *ABCJ*. The coordinate system *x*_1_*O*_1_*y*_1_ is established by assuming that point *A* is the origin of the coordinate system and *AJ* is the positive direction of the *x*-axis. An *ABCJ* crank–rocker mechanism is formed when *AB* rotates through a complete cycle in the coordinate system *x*_1_*O*_1_*y*_1_. Particularly, the four links *JA*, *AB*, *BC*, and *CJ* are represented by *L*_1_, *L*_2_, *L*_3_, and *L*_4_, respectively, while the lengths of the four links *AJ*, *AB*, *BC*, and *CJ* are represented by *l*_1_, *l*_2_, and *l*_3_. The angles between *L*_2_, *L*_3_, and *L*_4_ and the positive direction of the *x*-axis in the coordinate system *x*_1_*O*_1_*y*_1_ are denoted by *θ*_2_, *θ*_3_, and *θ*_4_, respectively. The angular velocities of the motions of *L*_2_, *L*_3_, and *L*_4_ are denoted by *ω*_2_, *ω*_3_, and *ω*_4_, respectively. The angular accelerations of the motions of *L*_2_, *L*_3_, and *L*_4_ are denoted by *α*_2_, *α*_3_, and *α*_4_, respectively. The closed vector equations are obtained using vector methods as follows:

(1) $$\vec{L_{2}}+\vec{L_{3}}=\vec{L_{1}}+\vec{L_{4}}$$

The plural form is as follows:

(2) $$l_{2}e^{i\theta_{2}}+l_{3}e^{i\theta_{3}}=l_{1}e^{i\theta_{1}}+l_{4}e^{i\theta_{4}}$$

In the coordinate system *x*_1_*O*_1_*y*_1_, *L*_1_ coincides with the positive direction of the *x*-axis, so *θ*_1_ = 0. Applying Euler’s formula *e^iθ^* = cos*θ* + *i*sin*θ*, the nonlinear equation system is obtained:

(3) $$\left[\begin{array}{cc} \cos\theta_{2} & \cos\theta_{3}\\ \sin\theta_{2} & \sin\theta_{3} \end{array}\right]\left[\begin{array}{c} l_{2}\\ l_{3} \end{array}\right]=\left[\begin{array}{cc} 1 & \cos\theta_{4}\\ 0 & \sin\theta_{4} \end{array}\right]\left[\begin{array}{c} l_{1}\\ l_{4} \end{array}\right]$$

Based on *θ*_n_ = *ω*_n_*t*, by differentiating with respect to time *t*, the angular velocity and angular acceleration of the *BC* rod and *CD* rod can be solved:

(4) $$\left[\begin{array}{cc} \omega_{2}\sin\theta_{2} & \omega_{3}\sin\theta_{3}\\ \omega_{2}\cos\theta_{2} & \omega_{3}\cos\theta_{3} \end{array}\right]\left[\begin{array}{c} l_{2}\\ l_{3} \end{array}\right]=\left[\begin{array}{c} \omega_{4}\sin\theta_{4}\\ \omega_{4}\cos\theta_{4} \end{array}\right]l_{4}$$

(5) $$\left[\begin{array}{cc} {\omega_{2}}^{2}\cos\theta_{2}+\alpha_{2}\sin\theta_{2} & {\omega_{3}}^{2}\cos\theta_{3}+\alpha_{3}\sin\theta_{3}\\ {\omega_{2}}^{2}\sin\theta_{2}-\alpha_{2}\cos\theta_{2} & {\omega_{3}}^{2}\sin\theta_{3}-\alpha_{3}\cos\theta_{3} \end{array}\right]\left[\begin{array}{c} l_{2}\\ l_{3} \end{array}\right]=\left[\begin{array}{c} {\omega_{4}}^{2}\cos\theta_{4}+\alpha_{4}\sin\theta_{4}\\ {\omega_{4}}^{2}\sin\theta_{4}-\alpha_{4}\cos\theta_{4} \end{array}\right]l_{4}$$

(6) $$\omega_{3}=\frac{\omega_{2}l_{2}\sin\left(\theta_{2}-\theta_{4}\right)}{l_{3}\sin\left(\theta_{4}-\theta_{3}\right)}$$

(7) $$\omega_{4}=\frac{\omega_{2}l_{2}\sin\left(\theta_{2}-\theta_{3}\right)}{l_{4}\sin\left(\theta_{4}-\theta_{3}\right)}$$

(8) $$\alpha_{3}=\frac{\omega_{2}^{2}l_{2}\cos(\theta_{2}-\theta_{4})-\omega_{3}^{2}l_{3}\cos(\theta_{3}-\theta_{4})+\omega_{4}^{2}l_{4}}{l_{3}\sin(\theta_{4}-\theta_{3})}$$

(9) $$\alpha_{4}=\frac{\omega_{2}^{2}l_{2}\cos(\theta_{2}-\theta_{3})-\omega_{4}^{2}l_{4}\cos(\theta_{4}-\theta_{3})+\omega_{3}^{2}l_{3}}{l_{4}\sin(\theta_{4}-\theta_{3})}$$

The theoretical configuration of the second-level mechanism of the bionic bullfrog hindlimb is a four-bar mechanism composed of *JDHI*. With point *J* as the coordinate origin, a coordinate system *x*_2_*O*_2_*y*_2_ is established, with *JI* as the positive direction of the *x*-axis. The coordinates of the origin *J* in the *x*_1_*O*_1_*y*_1_ coordinate system are (*l*_1_,0). The *x*-axis of the *x*_2_*O*_2_*y*_2_ coordinate system is rotated by angle ∠2 = *β* relative to the *x*-axis of the *x*_1_*O*_1_*y*_1_ coordinate system.

Based on the translation coordinates and rotation angle, the rotation matrix *R*, translation matrix *T*, and transformation matrix *M* can be obtained:

(10) $$R_{2}=\left(\begin{array}{ccc} \cos\beta & -\sin\beta & 0\\ \sin\beta & \cos\beta & 0\\ 0 & 0 & 1 \end{array}\right)$$

(11) $$T_{2}=\left(\begin{array}{ccc} 1 & 0 & l_{1}\\ 0 & 1 & 0\\ 0 & 0 & 1 \end{array}\right)$$

(12) $$M_{2}=R_{2}T_{2}=\left(\begin{array}{ccc} \cos\beta & -\sin\beta & l_{1}\\ \sin\beta & \cos\beta & 0\\ 0 & 0 & 1 \end{array}\right)$$

Since members *CJ* and *JD* are rigidly fixed, the velocity of member *JD* in the coordinate system *x*_2_*O*_2_*y*_2_ is as follows:

(13) $$\omega_{6}=\omega_{4}+\Delta \omega_{2}$$

In the coordinate system *x*_2_*O*_2_*y*_2_, *JD* acts as the active member, oscillating back and forth to form the *JDHI* double-rocker mechanism. Specifically, *L*_5_, *L*_6_, *L*_7_, and *L*_8_ correspond to the four links *IJ*, *JD*, *DH*, and *HI*, respectively, while *l*_5_, *l*_6_, *l*_7_, and *l*_8_ correspond to the lengths of these four links. *θ*_6_, *θ*_7_, and *θ*_8_ represent the angles between *L*_6_, *L*_7_, and *L*_8_ and the positive direction of the *x*-axis in the *x*_2_*O*_2_*y*_2_ coordinate system. *ω*_6_, *ω*_7_, and *ω*_8_ represent the angular velocities of *L*_6_, *L*_7_, and *L*_8_, respectively. *α*_6_, *α*_7_, and *α*_8_ represent the angular accelerations of *L*_6_, *L*_7_, and *L*_8_, respectively. Using the vector method to establish closed vector equations for calculation, the angular velocities of members *DH* and *HI* in the *x*_1_*O*_1_*y*_1_ coordinate system can be calculated through the same computational process:

(14) $$\omega_{8}=\frac{\omega_{6}l_{6}\sin\left(\theta_{6}-\theta_{7}\right)}{l_{8}\sin\left(\theta_{8}-\theta_{7}\right)}=\frac{\left(\omega_{4}+\Delta \omega_{2}\right)l_{6}\sin\left(\theta_{6}-\theta_{7}\right)}{l_{8}\sin\left(\theta_{8}-\theta_{7}\right)}-\Delta \omega_{2}$$

(15) $$\omega_{7}=\frac{\omega_{6}l_{6}\sin\left(\theta_{6}-\theta_{7}\right)}{l_{7}\sin\left(\theta_{8}-\theta_{7}\right)}=\frac{\left(\omega_{4}+\Delta \omega_{2}\right)l_{7}\sin\left(\theta_{6}-\theta_{7}\right)}{l_{8}\sin\left(\theta_{8}-\theta_{7}\right)}-\Delta \omega_{2}$$

(16) $$\alpha_{8}=\frac{{\left(\omega_{4}+\Delta \omega_{2}\right)}^{2}l_{6}\cos(\theta_{6}-\theta_{7})-\omega_{8}^{2}l_{8}\cos(\theta_{8}-\theta_{7})+\omega_{7}^{2}l_{7}}{l_{8}\sin(\theta_{8}-\theta_{7})}$$

(17) $$\alpha_{7}=\frac{-{\left(\omega_{4}+\Delta \omega_{2}\right)}^{2}l_{6}\cos(\theta_{6}-\theta_{8})-\omega_{7}^{2}l_{7}\cos(\theta_{7}-\theta_{8})+\omega_{8}^{2}l_{8}}{l_{7}\sin(\theta_{7}-\theta_{8})}$$

In the third-level mechanism of the bionic bullfrog hind limb, *DE* is regarded as a fixed frame, with *D* as the coordinate origin and *DE* as the positive direction of the *x*-axis for establishing the transformed coordinate system *x*_3_*O*_3_*y*_3_. Since the rod *DE* is rigidly fixed to the rod *CJ* and ∠*EDJ* = *φ*, the transformation parameters of the transformed coordinate system can be obtained:

(18) $$\mathrm{translation}\;\mathrm{coordinates}:\;\left(l_{1}+l_{6}\cos\left(\omega_{6}t+\beta \right),l_{6}\sin\left(\omega_{6}t+\beta \right)\right)$$

(19) $$\mathrm{rotation}\;\mathrm{angle}:\;∠3=\varphi -\left(\pi -\left(\omega_{6}t+\beta \right)\right)$$

Similarly, based on the translation coordinates and rotation angle, the rotation matrix *R*, translation matrix *T*, and transformation matrix *M* can be obtained:

(20) $$R_{3}=\left(\begin{array}{ccc} \cos\left(\varphi -\left(\pi -\left(\omega_{6}t+\beta \right)\right)\right) & -\sin\left(\varphi -\left(\pi -\left(\omega_{6}t+\beta \right)\right)\right) & 0\\ \sin\left(\varphi -\left(\pi -\left(\omega_{6}t+\beta \right)\right)\right) & \cos\left(\varphi -\left(\pi -\left(\omega_{6}t+\beta \right)\right)\right) & 0\\ 0 & 0 & 1 \end{array}\right)$$

(21) $$T_{3}=\left(\begin{array}{ccc} 1 & 0 & l_{1}+l_{6}\cos\left(\omega_{6}t+\beta \right)\\ 0 & 1 & l_{6}\sin\left(\omega_{6}t+\beta \right)\\ 0 & 0 & 1 \end{array}\right)$$

(22) $$M_{3}=\left(\begin{array}{ccc} \cos\left(\varphi -\left(\pi -\left(\omega_{6}t+\beta \right)\right)\right) & -\sin\left(\varphi -\left(\pi -\left(\omega_{6}t+\beta \right)\right)\right) & l_{1}+l_{6}\cos\left(\omega_{6}t+\beta \right)\\ \sin\left(\varphi -\left(\pi -\left(\omega_{6}t+\beta \right)\right)\right) & \cos\left(\varphi -\left(\pi -\left(\omega_{6}t+\beta \right)\right)\right) & l_{6}\sin\left(\omega_{6}t+\beta \right)\\ 0 & 0 & 1 \end{array}\right)$$

Since members *DG* and *DH* are rigidly fixed, the velocity of member *DG* in the coordinate system *x*_2_*O*_2_*y*_2_ is as follows:

(23) $$\omega_{12}=\omega_{7}+\Delta \omega_{3}$$

The theoretical configuration of the third-level mechanism of the bionic bullfrog hindlimb is a four-bar mechanism composed of *DEFG*. With point *D* as the origin, a coordinate system *x*_3_*O*_3_*y*_3_ is established with *DE* as the positive direction of the x-axis. In the coordinate system *x*_3_*O*_3_*y*_3_, *DG* serves as an active component, oscillating back and forth to form a *DEFG* double-rocker mechanism. Specifically, *L*_9_, *L*_10_, *L*_11_, and *L*_12_ correspond to the four links *DE*, *EF*, *FG*, and *GD*, respectively, and *l*_9_, *l*_10_, *l*_11_, and *l*_12_ correspond to the lengths of the four links *DE*, *EF*, *FG*, and *GD*, respectively. *θ*_10_, *θ*_11_, and *θ*_12_ represent the angles between *L*_10_, *L*_11_, and *L*_12_ and the positive direction of the x-axis of the *x*_3_*O*_3_*y*_3_ coordinate system. *ω*_10_, *ω*_11_, and *ω*_12_ represent the angular velocities of the motion of *L*_10_, *L*_11_, and *L*_12_. *α*_10_, *α*_11_, and *α*_12_ represent the angular accelerations of the movements of *L*_10_, *L*_11_, and *L*_12_, respectively. Using vector methods to establish closed vector equations for calculation, the angular velocities of members *EF* and *FG* in the *x*_1_*O*_1_*y*_1_ coordinate system can be calculated through the same computational process:

(24) $$\omega_{11}=\frac{\left(\frac{\left(\omega_{4}+\Delta \omega_{2}\right)l_{7}\sin\left(\theta_{6}-\theta_{7}\right)}{l_{8}\sin\left(\theta_{8}-\theta_{7}\right)}-\Delta \omega_{2}+\Delta \omega_{3}\right)l_{12}\sin\left(\theta_{12}-\theta_{10}\right)}{l_{11}\sin\left(\theta_{11}-\theta_{10}\right)}-\Delta \omega_{3}$$

(25) $$\omega_{10}=\frac{\left(\frac{\left(\omega_{4}+\Delta \omega_{2}\right)l_{7}\sin\left(\theta_{6}-\theta_{7}\right)}{l_{8}\sin\left(\theta_{8}-\theta_{7}\right)}-\Delta \omega_{2}+\Delta \omega_{3}\right)l_{12}\sin\left(\theta_{12}-\theta_{11}\right)}{l_{10}\sin\left(\theta_{10}-\theta_{11}\right)}-\Delta \omega_{3}$$

(26) $$\alpha_{11}=\frac{{\left(\frac{\left(\omega_{4}+\Delta \omega_{2}\right)l_{7}\sin\left(\theta_{6}-\theta_{7}\right)}{l_{8}\sin\left(\theta_{8}-\theta_{7}\right)}-\Delta \omega_{2}+\Delta \omega_{3}\right)}^{2}l_{12}\cos(\theta_{12}-\theta_{10})-\omega_{11}^{2}l_{11}\cos(\theta_{11}-\theta_{10})+\omega_{10}^{2}l_{10}}{l_{11}\sin(\theta_{11}-\theta_{10})}$$

(27) $$\alpha_{10}=\frac{{\left(\frac{\left(\omega_{4}+\Delta \omega_{2}\right)l_{7}\sin\left(\theta_{6}-\theta_{7}\right)}{l_{8}\sin\left(\theta_{8}-\theta_{7}\right)}-\Delta \omega_{2}+\Delta \omega_{3}\right)}^{2}l_{12}\cos(\theta_{12}-\theta_{11})-\omega_{10}^{2}l_{10}\cos(\theta_{10}-\theta_{11})+\omega_{11}^{2}l_{11}}{l_{10}\sin(\theta_{10}-\theta_{11})}$$

## 4. Bionic Bullfrog Experiment and Analysis

### 4.1. Experimental Prototype Design of a Bionic Bullfrog Hind Limb Propulsion System

**Figure 7:**
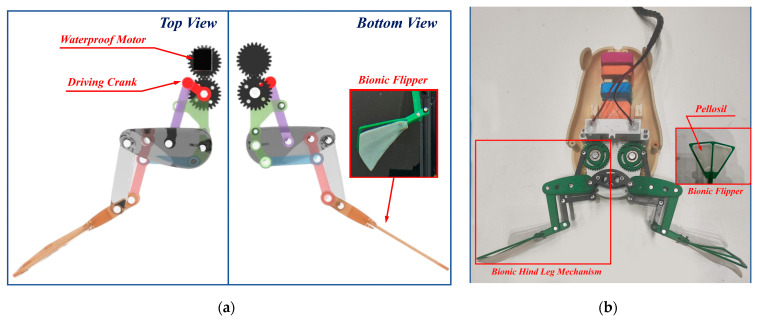
Bionic bullfrog mechanism: (**a**) rendering diagram of the bionic bullfrog mechanism; (**b**) bionic bullfrog mechanism physical map.

An experimental prototype design of the bionic bullfrog hindlimb was developed based on the theoretical design of the bionic bullfrog hindlimb propulsion mechanism. SolidWorks 2021 software was employed to generate the three-dimensional model of the bionic bullfrog hindlimb propulsion mechanism. As shown in Figure 7a, the bionic bullfrog hindlimb propulsion mechanism is powered by two symmetrical waterproof motors directly connected to gears. The driving crank of the bionic bullfrog hindlimb propulsion mechanism rotates continuously for a full cycle through gear transmission, allowing the entire mechanism to cycle through leg strikes along a predefined trajectory. During the cyclic kicking process, the hind appendages of bullfrogs enhance forward propulsion force and decrease backward resistance in nature. This is accomplished through the movement of the phalanges, which enables the flipper to open when the hind limbs are extended, thereby increasing the effective area, and to close when the hind limbs revert to a contracted state, thereby decreasing the effective area. Figure 7b is a physical image of the bionic bullfrog’s hind leg that has been installed.

### 4.2. Motion Capture Experiment on the Hind Legs of a Bionic Bullfrog

**Figure 8:**
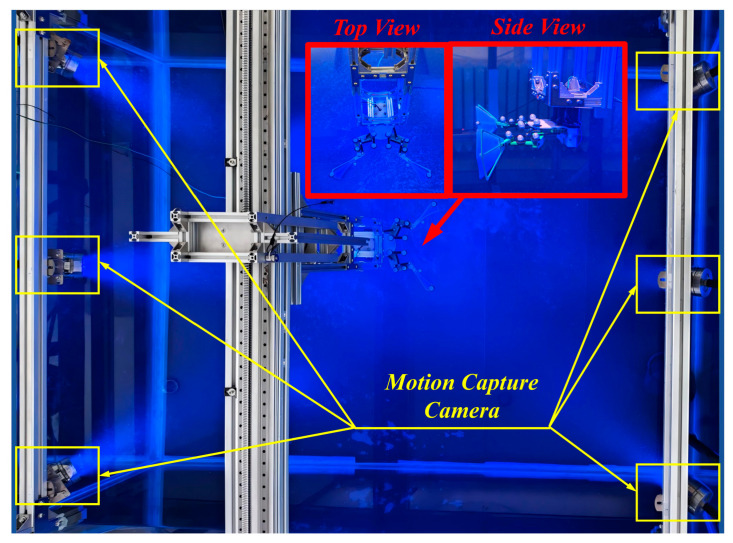
Motion capture experiment diagram of the bionic bullfrog hind leg mechanism.

The bionic bullfrog hindlimb propulsion system’s motion performance was evaluated using the same experimental apparatus as in the real bullfrog motion capture experiments. The complex parts were printed using FDM technology on a 3D printer, which was based on three-dimensional modeling. The FDM process utilizes a fused deposition modeling technique that is applied layer by layer, thereby facilitating the precise integrated manufacturing of nonlinear structures within biomimetic mechanisms and satisfying the high-precision requirements for link motion trajectories [32]. Polycarbonate sheets (PC sheets) were used as raw materials, and a micro milling machine was employed to carve simple planar parts from three-dimensional models [33], ensuring that part dimensional tolerances met the assembly requirements of the mechanism. PC material maintains structural integrity in underwater experimental environments by combining water resistance and impact resistance. Pins are employed to connect components at each rotating joint, thereby guaranteeing the linkage mechanism’s rotational freedom. The biomimetic hindlimb’s kicking and recuperation motions are guaranteed to be continuous and repeatable using split pins to secure these, thereby reducing movement gaps. The experimental prototype of the bionic bullfrog hind limb propulsion system is affixed to the extremity of the movable experimental object fixation frame in the motion capture experimental platform, as shown in Figure 8. Five motion capture spheres are affixed to each bionic bullfrog hind limb, utilizing the same points as in the motion capture experiment with a real bullfrog.

**Figure 9:**
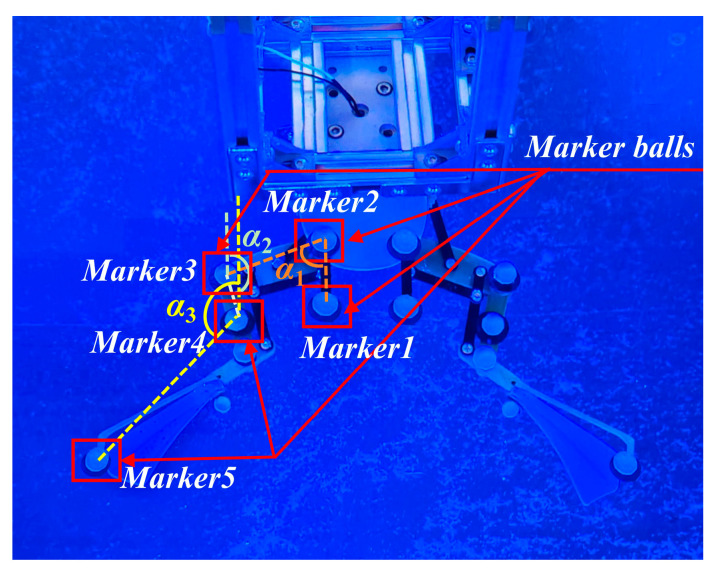
Schematic diagram of marking points and measurement angles on the hind legs of a bionic bullfrog.

**Figure 10:**
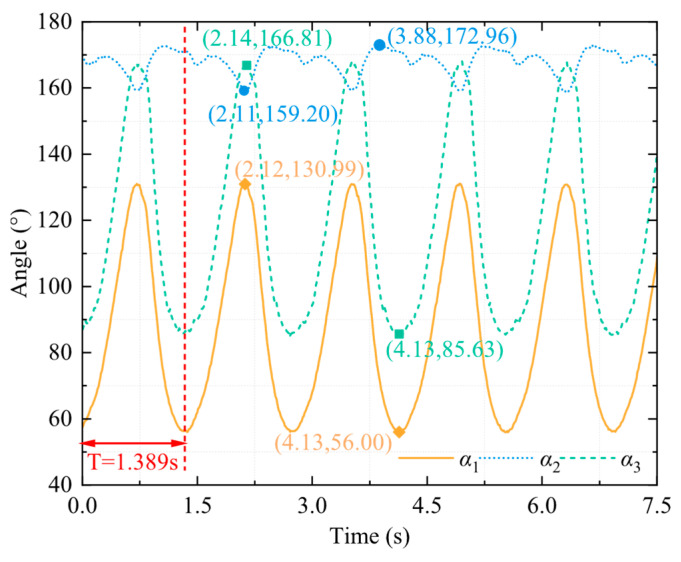
Motion capture data chart for the bionic bullfrog.

Two motors drove the bionic bullfrog’s hindlimb at the start of the experiment. NOKOV underwater motion capture cameras were used to record motion capture data, which were then transmitted to a computer. By repeatedly performing flexion and extension movements of the bionic bullfrog hindlimb propulsion system prototype, five sets of motion capture data were obtained to reduce random and systematic errors. The bionic hindlimb mechanism was driven by a constant voltage of 9 V on both sides. The motion capture system measured the angles *α*_1_, *α*_2_, and *α*_3_ between the thigh, lower leg, and webbed foot of the biomimetic hindlimb mechanism relative to the vertical direction of the body in real time, as shown in Figure 9. Analysis revealed the changes in the three angles *α*_1_, *α*_2_, and *α*_3_ during the frog’s hindlimb movement, as shown in Figure 10. According to the analysis, the movement cycle of the bionic bullfrog is 1.389 s, slightly shorter than the real bullfrog’s movement cycle. The range of variation in angle *α*_1_ between the bullfrog’s thigh and the vertical direction of the bullfrog’s body is approximately 56° to 130.99°. The peak standard deviation is ±2.3°, and the valley standard deviation is ±1.8°. The range of variation for angle *α*_2_, the angle between the calf and the vertical direction of the frog’s body, is approximately 159.2° to 172.96°. The peak standard deviation is ±1.5°, and the valley standard deviation is ±1.1°. The range of variation for angle *α*_3_, the angle between the webbed foot and the vertical direction of the frog’s body, is approximately 85.63° to 166.81°. The peak standard deviation is ±1.2°, and the valley standard deviation is ±0.9°.

**Figure 11:**
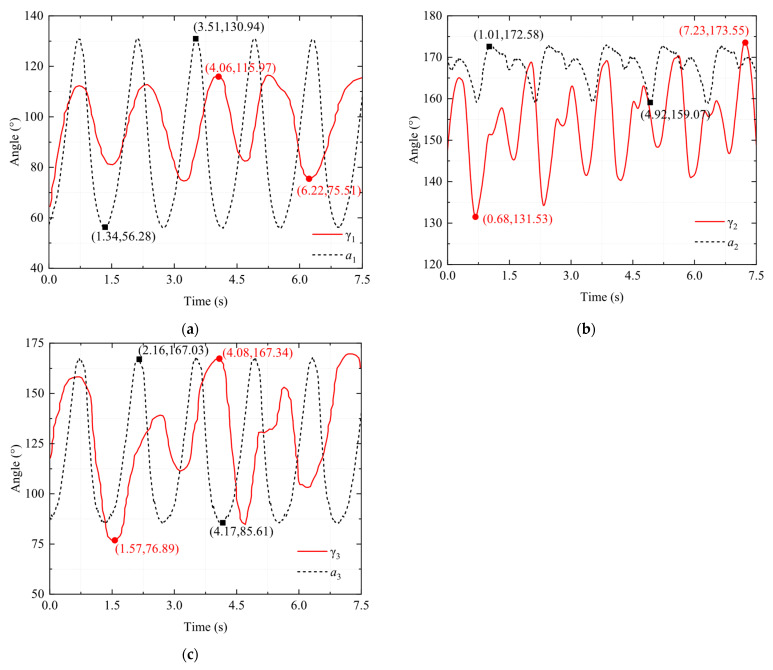
Comparison of motion capture data for the hind legs of the real bullfrog and the bionic bullfrog: (**a**) comparison of thigh angle changes; (**b**) comparison of lower leg angle changes; (**c**) comparison of webbed foot angle changes.

Figure 11 shows a comparison of the corresponding data for these three angles between the bionic bullfrog and the real bullfrog. Due to the inability to fully control the movement cycles of the bionic frog and the real frog, achieving complete consistency is challenging. As a result, visual differences in the images may be exaggerated. The range of joint angle changes in the hindlimbs of the bionic bullfrog may be influenced by factors such as link length and output voltage. Figure 11a shows the data for the thigh angle *γ*_1_ of the real bullfrog and the thigh angle *α*_1_ of the bionic bullfrog as a function of time. Analysis indicates that the biomimetic mechanism has a wider range of angular changes, with a lower limit and a higher upper limit. This is because the thigh muscles of the real bullfrog are contracted under neural control and restricted by soft tissues such as ligaments and tendons, while the bionic bullfrog can achieve a larger angle during the thigh extension phase through mechanical drive. Additionally, the multi-link design enhances the swing angle through a cumulative effect. However, the angular change trends of both systems exhibit similarity, following a pattern of initial increase followed by decrease within a single cycle, consistent with the periodic characteristic of “angle increase during the propulsion phase–angle decrease during the recovery phase.” Figure 11b compares the time-varying data of the real bullfrog thigh angle *γ*_2_ with the bionic frog thigh angle *α*_2_. The changing angle exhibits a “rise–fall–rise–fall” fluctuation within one cycle, directly reflecting the sub-phases of its “kick–retract” action, corresponding to the four sub-phases of “pre-kick extension–powerful kick extension–pre-retraction–powerful recovery” four sub-phases: During the pre-kick extension phase, kinetic energy is stored, and the calf angle initially increases; during the powerful kick extension phase, due to the explosive contraction of muscles and the buffering effect of water resistance, the angle first temporarily decreases and then reaches a peak; the pre-retraction phase facilitates a smooth transition from “expansion” to “contraction” of the webbed foot, with the angle temporarily increasing again to reduce water flow disturbance; the powerful recovery phase rapidly returns to the initial state, with the angle decreasing again to the initial position. During the propulsion phase, the upper limit value of *α*_2_ is slightly higher than *γ*_2_, as the mechanical linkage achieves a more near-180° extension through rigid transmission, thereby enhancing the stability of thrust output; during the recovery phase, the lower limit of *α*_2_ is greater than *γ*_2_, as the mechanical structure lacks the soft tissue friction resistance of biological tissues, requiring a slightly larger angle to ensure the continuity of link movement. However, both angle curves exhibit a synchronized trend of “propulsion extension–recovery flexion,” confirming the biomimetic mechanism’s reproduction of the biological lower leg movement sequence. The biomimetic mechanism achieves the core function of actively folding the lower limb during the recovery phase to reduce resistance. The analysis results validate the simulation of the frog’s knee joint full extension by the three-stage four-bar mechanism, indicating that the extension angle of the biomimetic lower limb is closer to 180°. The excessive angle during the flexion phase may be influenced by the motion trajectory of the link mechanism. Figure 11c shows a comparison of the changes in the angle *α*_3_ of the bionic bullfrog’s webbed feet and the angle *γ*_3_ of the real bullfrog’s webbed feet over time. Analysis indicates that the ranges of angular changes between the two are highly similar. This is attributed to the biomimetic mechanism employing a single-sided skeletal silicone webbing design, which increases the effective area by tightly adhering the webbing to the skeleton during the kicking phase and reduces resistance by separating from the skeleton due to water resistance during the recovery phase. This mechanism accurately simulates the opening and closing mechanism of the real bullfrog’s webbed membrane. The elasticity of the webbed membrane and the range of motion of the toe joints are limiting factors for the real bullfrog’s webbed feet. However, the biomimetic mechanism, achieved through a three-degree-of-freedom four-bar linkage mechanism design, enables the webbed feet to reach a maximum opening angle comparable to the biological prototype during the final stage of propulsion. The angle curves of both systems exhibit synchronous periodic fluctuations, with angles increasing during the propulsion phase and decreasing during the recovery phase. During the propulsion phase, the angle increases, reflecting the explosive movement characteristics of kicking force. During the recovery phase, the angle decreases, simulating the biological habit of folding limbs to reduce resistance.

By comparing the angles of the three sets of curves, it was found that the angle changes in the hindlimb movements of the bionic bullfrog and the real frog exhibit a certain degree of similarity, primarily in the trend of angle changes. This indicates that the hindlimbs of the bionic bullfrog can achieve swimming movements similar to those of the real frog; however, there are differences in the range of angle changes and the movement cycle. The reasons for these differences include the limited number of real frog samples, significant uncontrollable errors during data collection, and the fixed voltage applied to the bionic bullfrog’s drive system.

### 4.3. Six-Dimensional Force Experiment on the Hind Legs of a Bionic Bullfrog

**Figure 12:**
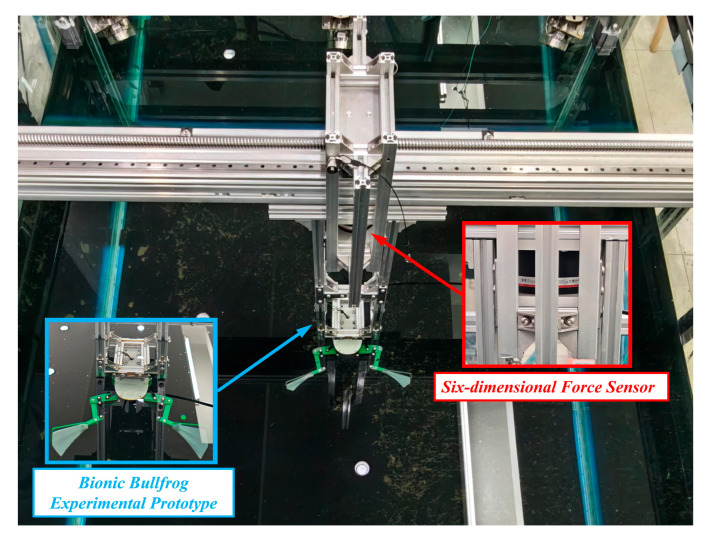
Experimental diagram of six-dimensional force on the hind legs of a bionic bullfrog.

**Figure 13:**
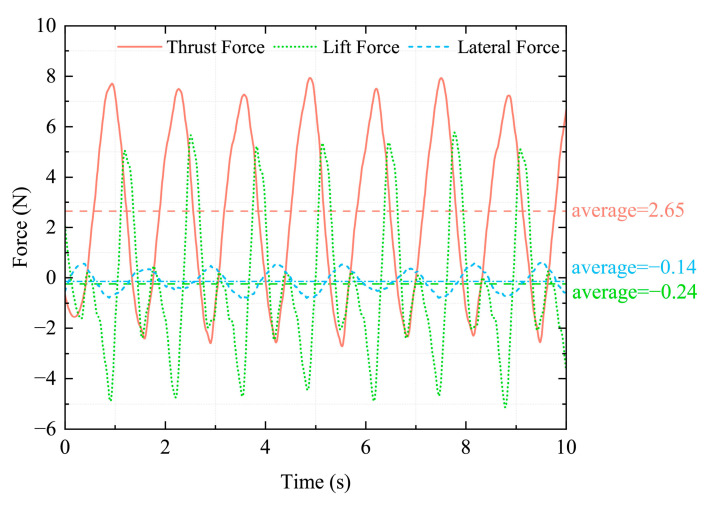
Bionic bullfrog hind leg propulsion six-dimensional force data chart.

To further evaluate the propulsion performance of the bionic bullfrog hindlimb propulsion system, a six-degree-of-freedom force sensor was employed to conduct experiments on the experimental prototype following the completion of the motion capture experiment, as illustrated in Figure 12. The experimental prototype’s original motion capture balls were removed prior to the experiment to prevent their impact on the data. The movable experimental object fixation frame on the experimental platform was replaced with a support that was rigidly connected to the robot and outfitted with a six-degree-of-freedom sensor. The bionic bullfrog’s hindlimbs were propelled by two motors that were maintained at a constant voltage of 9 V at the commencement of the experiment. The motion data were recorded by the DECENT six-dimensional force sensor and transmitted to the computer. To mitigate systematic and random errors, five sets of corresponding six-dimensional force data were acquired by repeatedly executing the flexion and extension movements of the bionic bullfrog hindlimb propulsion system prototype. The data analysis and curve plotting were conducted to determine the variations in thrust, lateral force, and lift of the hindlimb propulsion system by averaging the five sets of experimental data, as illustrated in Figure 13. To reduce high-frequency noise, such as mechanical vibrations or environmental interference, a second-order Butterworth low-pass filter with a cutoff frequency of 30 Hz was used in the experiment to filter out high-frequency noise. The data were then further smoothed using a moving average filter with 15 sampling points. The continuity and reliability of the force curve were ensured by the sensor’s accuracy of 0.1% F.S., the removal of outliers using the 3σ criterion, and periodic overlap verification. The deviation in the peak-to-valley difference between adjacent periods is <5%, and the average period deviation across five repeated experiments is <0.02 s. Periodic variations are observed in all three forces. The analysis demonstrated that the biomimetic hindlimbs can generate positive thrust when both sides are moved simultaneously. The average thrust is 2.65 N, with a standard deviation of ±0.32 N. The maximum instantaneous thrust is 7.91 N, with a standard deviation of ±0.57 N. During movement, the yaw force is small, fluctuating around 0, with an average yaw force of 0.14 N, indicating that the robot can achieve smooth forward movement during synchronized propulsion of both hind legs. The webbed membrane is entirely extended during the kicking phase, with a single-sided skeleton supporting the silicone webbing. The webbed membrane generates an upward lift as water flows more rapidly on the upper surface and more slowly on the lower surface, resulting in an instantaneous maximum lift of approximately 5.66 N during the final stage of recovery or the initial stage of the subsequent striking phase. The webbed membrane separates from the single-sided skeleton during recovery because of water resistance. The membrane’s folding induces turbulence on the lower surface. The pressure on the upper surface is greater than that on the lower surface, resulting in a downward negative lift, and the maximum instantaneous lift is 5.12 N. The average lift throughout the entire movement process is 0.24 N, indicating that the vertical forces are largely balanced during the bionic frog leg movement.

**Figure 14:**
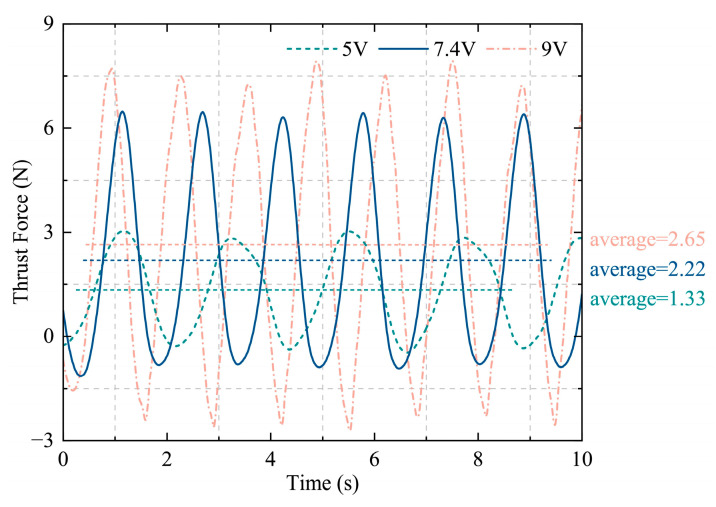
Comparison of thrust data for the hind legs of a bionic bullfrog under three different voltages.

The drive voltage is a critical parameter in the design of a bionic bullfrog hindlimb propulsion system, as it directly influences the propulsive force and movement frequency of the biomimetic hindlimb propulsion mechanism. This study conducted thrust comparison experiments under various voltages to investigate the correlation between drive voltage and thrust output, thereby further optimizing the power efficiency of the propulsion system, after verifying the basic six-dimensional force performance of the biomimetic hindlimb. The power supply module was replaced with an adjustable regulated power supply, while the six-degree-of-freedom force testing system was maintained. The bionic bullfrog hindlimb experimental prototype was maintained in its original state to guarantee that voltage was the only variable. The voltage gradient was established as follows: 5 V, 7.4 V, and 9 V. Five tests were conducted for each voltage condition, with the average value being taken to minimize error. Figure 14 presents the thrust fluctuations that occur under the three voltage scenarios. The average thrust value was obtained over a complete cycle. Analysis indicates that the average thrust of the biomimetic hindlimb propulsion system under a constant voltage of 5 V is 1.33 N, with a standard deviation of ±0.21 N. Under a constant voltage of 7.4 V, the average thrust is 2.22 N, with a standard deviation of ±0.28 N. Under a constant voltage of 9 V, the average thrust is 2.65 N, with a standard deviation of ±0.32 N. With an increase in voltage, the motor speed accelerates, the movement cycle shortens, and the effective area of the water flow and the speed of water pushed by the oscillating membrane are optimized synergistically, resulting in a nonlinear increase in thrust. This confirms the feasibility of optimizing dynamic thrust through voltage regulation.

## 5. Conclusions

This research presents a biomimetic underwater propulsion system design inspired by the locomotion mechanism of the bullfrog’s hind limbs. A three-stage four-bar linkage system simulates the bullfrog’s “kicking–recovery” motion, with the prototype constructed utilizing FDM 3D printing and PC board engraving techniques. Experimental results from motion capture studies comparing the real bullfrog and the bionic bullfrog hindlimbs reveal that the angular ranges of the biomimetic mechanism’s thigh, lower leg, and webbed foot are 56°–130.99°, 159.2°–172.96°, and 85.63°–166.81°, respectively. The calf extension angle approaches the bionic bullfrog’s physiological limit of 180°, while the webbed foot movement aligns with the biological prototype with an impressive accuracy of 98.5%, thereby fully validating the multi-link mechanism’s accurate simulation of biological kinematic characteristics. Six-dimensional force testing indicates that, under a 9V voltage drive, the biomimetic system produces an average thrust of 2.65 N and a peak instantaneous thrust of 7.91 N. The lift varies between +5.66 N and −5.12 N, whereas the vertical component of the resultant force remains predominantly balanced. The mean yaw force is merely 0.14 N, suggesting that coordinated propulsion of both hind limbs can achieve steady linear motion. Voltage gradient investigations further validated that as the input voltage escalated from 5 V to 9 V, the thrust demonstrated nonlinear augmentation, indicating a synergistic optimization process between motor velocity and fin membrane propulsion efficacy. This concept addresses the physiological constraints of biological muscles by mechanically enhancing joint movement ranges, offering underwater robots a propulsion solution characterized by minimal disturbance and great stability. The core conclusion has been verified as statistically significant. Nevertheless, the pronounced variations in lift throughout the propulsion and recovery phases may result in the robot demonstrating periodic vertical displacement. Future research will concentrate on incorporating guide channels or flexible baffles during the recovery phase to mitigate the impact of water flow on the folded flipper membrane through flow field guidance, or on integrating elastic components at the connection points of the flipper membrane to enhance its closed posture and diminish negative lift peaks. Additionally, future work will expand the sample size of repeated experiments, incorporate error bars in relevant plots to reflect data variability, and conduct more in-depth statistical analysis to further enhance the robustness and reliability of the research findings.

## Data Availability

The data presented in this study are available upon request from the corresponding author.
